# Preparation of a Ceramifiable Phenolic Foam and Its Ceramization Behavior

**DOI:** 10.3390/polym14081591

**Published:** 2022-04-14

**Authors:** Kaihong Tang, Yang Yu, Guiqiu Xu, Xiaojun Tang, Ailing Zhang, Tiejun Ge, Yongjiang Li

**Affiliations:** 1 Key Laboratory of Polymer and Catalyst Synthesis Technology of Liaoning Province, School of Environmental and Chemical Engineering, Shenyang University of Technology, Shenyang 110870, China; t_angkh@163.com (K.T.); lyj@ciac.ac.cn (Y.L.); 2 School of Materials Science and Engineering, Shenyang University of Chemical Technology, Shenyang 110142, China; yy_email@163.com (Y.Y.); 653x@163.com (G.X.); tangxj2008@yeah.net (X.T.); 3 Polymer Material Synthesis and Processing Professional Technology Innovation Center of Liaoning Province, Shenyang University of Chemical Technology, Shenyang 110142, China

**Keywords:** phenolic foam, low melting point glass frits, ceramization, flame retardancy, thermal stability

## Abstract

Ceramifiable phenolic foam (GC-PF) with a low ceramization temperature has been prepared by incorporation of low melting point glass frits (LMG) containing B_2_O_3_ and Na_2_O as main components into a phenolic resin matrix. Fourier transform infrared spectrometry, X-ray diffractometry, and scanning electron microscopy were used for assessment of the structure, phase composition, and morphology of GC-PF before and after combustion analysis, respectively. A glassy ceramic protective layer is formed when GC-PF is exposed to flame or a high temperature environment. The presence of LMG not only reduces the level of defects in the phenolic foam cell wall (gas escape pore), but also promotes the generation of a glassy ceramic protective layer that could inhibit heat feedback from the combustion zone and reduce the rate of formation of volatile fuel fragments. Thermogravimetric analysis and differential scanning calorimetry were used to establish that GC-PF exhibits excellent thermal stability. Limiting oxygen index (LOI) determination suggests that GC-PF displays good flame retardancy. The LOI of GC-PF was as high as 45.6%, and the char residue at 900 °C was six times greater than that for ordinary phenolic foam (O-PF). The area of the raw material matrix of GC-PF after combustion for 60 s was about 1.7 times larger than that for O-PF. A possible mode of formation of glassy ceramics has been proposed.

## 1. Introduction

Phenolic foam (PF) is an ideal thermal insulation material with high dimensional stability and low thermal conductivity [1,2,3]. It has been widely used in the thermal insulation of buildings and in aerospace applications due to its low cost and light weight [4]. However, high temperature combustion of PF material affords a low residue yield of loose char with little mechanical strength. This char tends to smolder after combustion is complete [5,6], and the flame retardancy of PF needs to be further improved. The flammability properties of phenolic foams may be improved by the presence of flame retardants [7,8,9,10,11] or modification of the molecular structure of the phenolic resin matrix [12,13,14,15]. However, problems associated with the formation of loose char and smoldering combustion remain.

Heat feedback from combustion of degradation volatiles may destroy the surface structure of the polymer, causing bulging or cracking [16]. When the ceramifiable polymer is exposed heat in a high temperature environment, the surface of the material can form a dense ceramic structure protective layer with a certain self-supporting strength [17,18,19,20]. A surface ceramic layer can effectively retard degradation of the internal structure of the material and reduce the generation of volatile fuel fragments to support combustion. Ceramicization of the material improves the strength of the char layer and prevents smoldering, which has important application value in the field of fire prevention and flame retardancy [16,21,22,23].

Phenolic resin-based ceramifiable blends represent a promising approach to the generation of phenolic materials of reduced flammability ceramic powder, which has been included in the preparation of composite laminates based on glass fibers and phenolic resin [24]. Ceramic phenolic resin matrix composites from boron-phenolic resin and ceramic fillers have been ceramized at 1000 °C [25,26]. Zirconia fiber hybrid alumina fiber/boron phenolic ceramifiable compositions have been ceramized at 1300 °C [27]. Novel phenolic/silica hybrid ceramic materials have been synthesized using a sol-gel method [28]. A novel preceramic polymer system based on silazane modified phenol formaldehyde resin has been reported [29]. The ceramization of MgO-Al_2_O_3_-SiO_2_/boron phenolic resin compositions under different sintering conditions have been studied [30]. Unfortunately, these phenolic compositions cannot be used to prepare foams. Further, the ceramic-forming temperature for these materials may be as high as 1000 °C. Ordinary phenolic foam (O-PF) is completely pyrolyzed at about 500 °C, which means that most ceramic-forming fillers are not active during the degradation process for PF. Therefore, it is necessary to develop a PF material that can undergo ceramization at a low temperature.

A ceramic structure can be formed by two methods: (1) softening of an amorphous fluxing agent at high temperature which adheres inorganic particles to form a continuous porous structure with excellent thermal stability [31,32,33]; (2) silica and siloxane macromolecules may be heated to form a silicon oxycarbide ceramic phase or directly used to connect inorganic particles [17,34,35,36]. At present, a difficulty for flame retardancy of ceramifiable polymer materials is that the ceramic-forming temperature of ceramic-forming fillers for rubber and resin is high (above 800 °C). Low-temperature ceramic-forming materials can be achieved by incorporation of ceramic fillers into polyolefins, but excessive addition of ceramic-forming fillers leads to a serious problem of flaming drip [37].

The softening temperature of low-melting glasses (glass fillers, glass frits) is lower than 800 °C. Low melting point glass frits (LMG) containing zinc phosphate, zinc borate, B_2_O_3_, alkaline salts, or other inorganic oxides can melt and tightly adhere high-melting inorganic particles at relatively low temperatures to form eutectic blends [33,38,39]. Subsequently, the eutectic blend reacts with an alkaline metal oxide or expansion system to form a glassy ceramic protective layer after crystallization [40,41]. This ceramization flame retardant method has been successfully applied to rubber composites [30,42]. An EVA composite prepared with ammonium polyphosphate/zinc borate as novel sintering aid forms a glassy ceramic layer containing α-Zn_3_(PO_4_)_2_ and orthophosphate at high temperature [43]. If this ceramization technology can be applied to PF, a PF material with a low ceramization temperature can be prepared.

Based on the flame retardant mode of action for ceramization, a ceramifiable phenolic foam (GC-PF) with a low ceramization temperature has been formed by incorporation of LMG containing B_2_O_3_ and Na_2_O as the main components into a phenolic resin matrix. The molecular structure and cell structure of the GC-PF were characterized, and the ceramization process of the foam was studied. A possible mode of formation of glassy ceramics has been proposed.

## 2. Materials and Methods

### 2.1. Materials

Phenol, paraformaldehyde, sodium hydroxide (as a deprotonation agent), n-pentane (as a blowing agent), and silane coupling agent (KH560) were purchased from the Tianjin Damao Chemical Reagent Factory (Tianjin, China). Tween-80 (as a surfactant) and sulfuric acid (98 wt.%, as a curing agent) were supplied by Sinopharm Chemical Reagent Co., Ltd. (Shanghai, China). Low melting point glass frits (LMG, having a softening point temperature at 450 °C) were provided by Foshan Jinggu Material Technology Co., Ltd. (Foshan, China). This LMG (grain size less than 5 μm) is a mix of oxides consisting of B_2_O_3_: 43.84% Na_2_O: 29.31% Bi_2_O_3_: 10.22% P_2_O_5_: 6.97% K_2_O: 4.01% Al_2_O_3_: 2.84% others: 2.81%.

### 2.2. Sample Preparation

#### 2.2.1. Synthesis of Phenolic Resin

Phenol and sodium hydroxide (1% of phenol weight) were placed into a three-necked flask containing a reflux condenser, a thermometer, and a stirring paddle. The three-necked flask was sealed and placed in a thermostat water bath (the temperature is about 80 °C) until the sodium hydroxide was completely dissolved. The paraformaldehyde (the molar ratio of paraformaldehyde to phenol is 1.9) was added into the flask evenly in six batches within half an hour. The temperature of the water bath was maintained for 35 min, and then the temperature was raised to 90 °C for 70–75 min. The resin was cooled to room temperature and sealed for 24 h for the preparation of PF.

#### 2.2.2. LMG Pretreatment

An ethanol aqueous solution (20 wt.%) dissolved with silane coupling agent (3% of LMG weight) was placed in a three-necked flask, sealed, and stirred in a 60 °C water bath for 30 min. LMG was added at a constant speed and stirred at 60 °C for 2 h. The activated LMG was dried in a vacuum drying oven (the temperature of the oven is about 80 °C) after being vacuum filtered.

#### 2.2.3. Preparation of GC-PF

Preparation formula of GC-PF is shown in Table 1. The phenolic resin and activated LMG were mechanically stirred evenly according to the formula. Surfactant and foaming agent were added sequentially for full stirring. A certain amount of curing agent was added and quickly stirred evenly. Following that, the resin was poured into the mold and placed in the oven at 78 °C for 30 min.

### 2.3. Measurements

The limiting oxygen index (LOI) was tested according to the Chinese National Standard (GB/T 2046-1993) with oxygen index tester (JF-3 Nanjing Analytical Instrument Factory Co., Ltd., Nanjing, China). Sample dimension was 15 × 1 × 1 cm^3^.

Dimension measurement of three sides of sample was guaranteed to be more than three positions, and the volume of sample was calculated by using average value. The mass of weighing sample was accurate to 0.5%, in grams (g). The apparent density was calculated using the following equation:

(1) $$\rho =\frac{m}{V},$$

where ρ (g/cm^3^) is the apparent density; m (g) is the mass of sample; V (cm^3^) is the volume of sample.

The bending strength was determined according to the Chinese National Standard (GB/T 8812-2007) and the compressive strength was determined according to the Chinese National Standard (GB/T 8813-2020) with RGL-type microcomputer control electronic universal testing machine (Shenzhen Rui Geer Instrument Co., Ltd., Shenzhen, China).

The surface morphology of PF after combustion was tested with a horizontal and vertical combustion instrument (TTech-GBT2408 TESTech Instrument Technologies Co., Ltd., Jiangsu, China), flame of height 150 mm, and a blue flame of height 20 ± 2 mm. The parameters are as follows: vertical combustion test sample size of length 125 ± 5 mm; width 13 ± 0.3 mm; and thickness of 3 ± 0.2 mm; tilt angle 20°, horizontal combustion sample size of width 25 ± 0.5 mm; and thickness of 20 ± 0.3 mm. Image analysis software (Photoshop CS6 and Image-pro plus 6.0) was used to analyze the morphology, map crack degree, and cell structure of the foam after combustion.

Fourier transform infrared spectrometer (FTIR) (NEXUS 470 Thermo Electron Corporation, Shanghai, China) was used to analyze the molecular structure of PF. The spectra were recorded in 400~4000 cm^−1^ range at a resolution of 4 cm^−1^ and a scanning number of 10.

The crystal phases of PF before and after combustion were identified by X-ray diffractometer (XRD) (D8 ADVANCE Bruker Daltonics Inc., Billerica, MA, USA). XRD data was obtained from 5~60° at a scan rate of 0.02°/s.

Scanning electron microscope (SEM) (SU8010 HITACHI, Tokyo, Japan) was used to observe the cell structure of PF before and after combustion. The surface of the sample was sprayed with gold before characterization.

A thermogravimetric (TG) analyzer (TG 209F1, Germany NETZSCH company, Selb, Germany) was used for comprehensive thermal analysis of PF under an air atmosphere at a temperature of 40~1000 °C and a heating rate of 10 °C/min.

The ceramization process of the GC-PF was studied using differential scanning calorimetry (DSC) (STA449C, Erich NETZSCH GmbH & Co. Holding KG, Selb, Germany) at a heating rate of 10 °C/min from 40 °C to 900 °C (air atmosphere, flow rate of 50 mL/min).

## 3. Results and Discussion

### 3.1. Apparent Density and LOI

**Figure 1:**
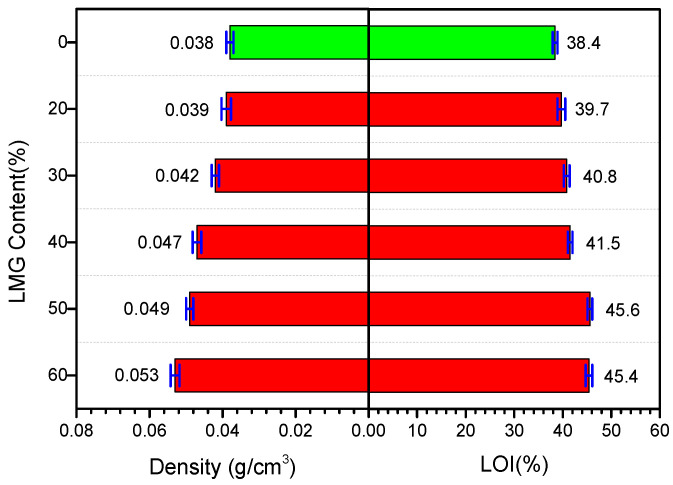
Apparent density and LOI of O-PF (green) and GC-PF (red).

As shown in Figure 1, both the apparent density and LOI of GC-PF increased with the increase of LMG content. As a lightweight foam, the apparent density of PF should be kept below 0.05 g/cm^3^. The addition of LMG resulted in an increase in the mass of GC-PF per unit volume. However, the apparent density was still lower than 0.05 g/cm^3^ when the LMG content was 50%. Compared with O-PF, the flame retardant property of GC-PF was significantly improved, and the LOI of GC-PF was as high as 45.6%. The addition of LMG not only increased inorganic flame retardant elements in raw material matrix, but also melted and adhered on the char layer surface during the foam combustion process to reduce the rate of generation of pyrolytic fuel fragments to support combustion.

### 3.2. Bending Strength and Compressive Strength

**Figure 2:**
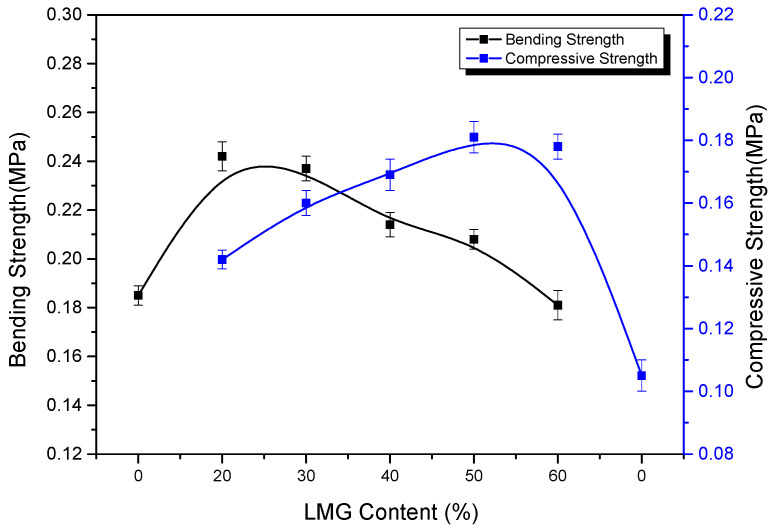
Effect of LMG addition on mechanical properties.

As shown in Figure 2, the bending strength of GC-PF was significantly improved when the LMG addition amount was 20%. This is because the addition of LMG can blunt the stress concentration at the crack tip when the foam bears the external force, thereby changing the direction and path of crack extension to consume more load energy [44,45]. However, excessive rigid particles in the foam increased the brittleness of GC-PF with increasing LMG content, resulting in a decrease in bending strength [24,46]. Although GC-PF exhibited better toughness at lower LMG content, there was no significant improvement in LOI. The bending strength of GC-PF was still higher than that of O-PF when the content of LMG was 50%. The compressive strength increases with the increase of LMG content, and the maximum compressive strength can reach 0.185 MPa when the LMG content is 50%. When the addition amount of LMG exceeded 50%, it was difficult to uniformly mix with resin matrix, and the compressive strength of GC-PF decreased. The mechanical properties, apparent density, and LOI test results showed that the comprehensive performance of GC-PF was the best when the LMG content was 50%.

### 3.3. Surface Morphology of Foam after Combustion

**Figure 3:**
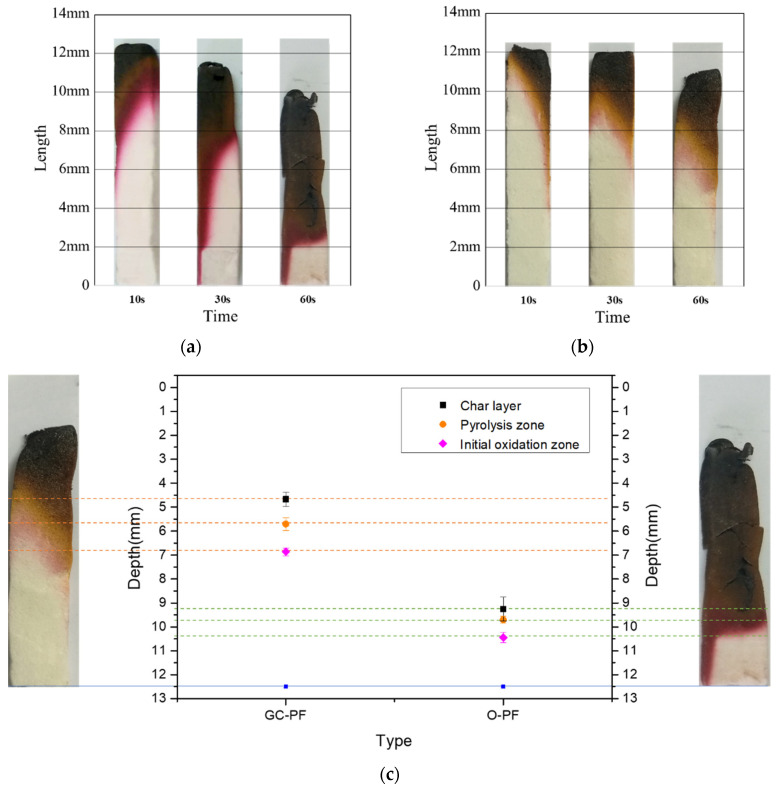
Surface morphology of PF after burning combustion: (**a**) O-PF with different combustion time; (**b**) GC-PF (LMG content 50%) with different combustion time; and (**c**) comparison of GC-PF and O-PF after combustion for 60 s.

Phenolic foams dehydrate rapidly during combustion and form a protective char layer. As shown in Figure 3a,b, GC-PF (LMG content 50%) and O-PF after combustion showed slight shrinkage, and the char layer gradually expanded with the increase of combustion time. GC-PF after combustion showed slight bending deformation, and O-PF after combustion basically retained its original shape. This may be due to the melting and interpenetration of inorganic oxides in GC-PF at high temperature, resulting in bending deformation of GC-PF after combustion [47]. Compared with O-PF, the char layer of GC-PF inhibited more heat under the same combustion conditions and time. The char layer strength of GC-PF after combustion was improved, and no obvious cracks and residues were observed. As shown in Figure 3c, the area of the raw material matrix of GC-PF after combustion for 60 s was about 1.7 times larger than that for O-PF. The reason may be that the presence of LMG can absorb part of heat during the combustion. Molten inorganic oxides adhere unmelted inorganic particles and the char generated by GC-PF together, which can reduce the rate of generation of pyrolytic fuel fragments to support and prevent the further spread of heat.

### 3.4. Ceramization of GC-PF

**Figure 4:**
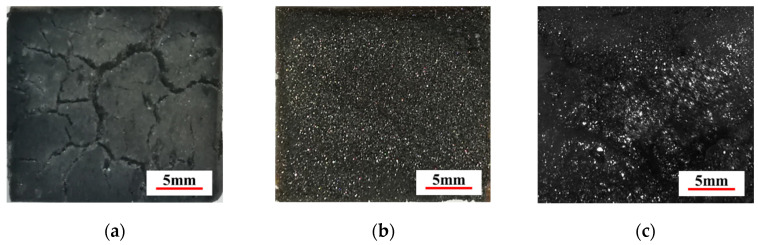
Char layer surface of phenolic foam after combustion: (**a**) O-PF; (**b**) GC-PF (LMG content 50%); and (**c**) ceramization of GC-PF.

Figure 4a,b are the char layer surfaces of O-PF and GC-PF (LMG content 50%) after combustion for 60 s under the same flame condition. A large amount of gas generated by pyrolysis of flammable substances (such as free formaldehyde, methylene, hydroxymethyl) contained in PF during the combustion process broke through cell walls and destroyed char layer structure of the foam. The char layer of O-PF had low strength, and the surface appeared map-cracks with many and deep crack branches. Uniform white particles appeared on the char layer surface of GC-PF after combustion for 60 s without obvious cracks. This may be due to the melting of LMG during foam combustion, which tightly sticks char and prevents the cracking of char layer. LMG with a short combustion time was resolidified into particles, which were uniformly distributed on the surface of char layer. It can be seen from Figure 4c that GC-PF formed a glassy ceramic protective layer on the surface of the char layer after a long combustion time. The glassy ceramic protective layer was formed by covering the surface of char layer with blends containing glassy borate and metaborate generated by B_2_O_3_ and Na_2_O (the main raw material of LMG), aryl configuration char of char layer, and unmelted inorganic particles. This layer can inhibit heat feedback from the combustion zone and reduce the rate of generation of pyrolytic fuel fragments to support combustion, and effectively protect the internal structure of the foam.

### 3.5. FTIR Analysis

**Figure 5:**
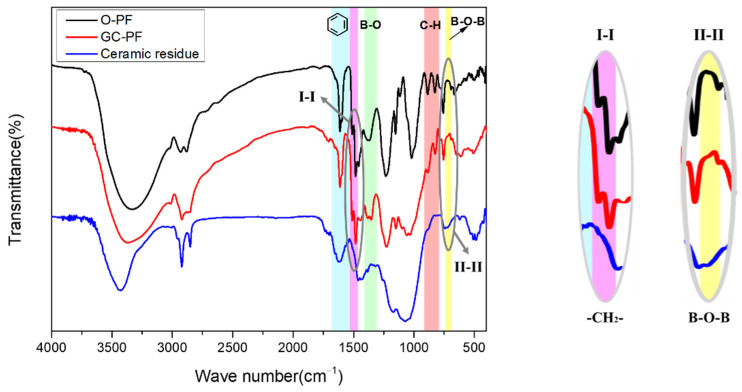
FTIR spectra of O-PF, GC-PF (LMG content 50%), and ceramic residue.

Figure 5 shows the FTIR spectra of O-PF, GC-PF (LMG content 50%), and ceramic residue. The peak at 1525~1660 cm^−1^ is assigned to the stretching vibration of benzene ring, the peak at 1463~1500 cm^−1^ is assigned to the bending vibration of -CH_2_- (connecting benzene ring), and the peak at 792~916 cm^−1^ is assigned to the bending vibration of C-H on the benzene ring. O-PF, GC-PF, and ceramic residue all contain benzene ring structures. The cleavage of -CH_2_- and C-H of PF during the combustion process resulted in the disappearance of the characteristic peaks at 1463~1525 cm^−1^ and 792~916 cm^−1^ of the ceramic residue. For the O-PF, the peak at 1310~1400 cm^−1^ is assigned to the characteristic peak of the -CH_2_-O-CH_2_-. For the GC-PF, the peak at 1322~1395 cm^−1^ is assigned to the characteristic peak of the B-O. Because B_2_O_3_ can react with Na_2_O to generate glassy borates during the combustion process of GC-PF, the characteristic peak (B-O) of ceramic residue disappears, and a new B-O-B characteristic peak appears at 681~737 cm^−1^. FTIR analysis verifies the structural changes of the GC-PF after combustion.

### 3.6. XRD Analysis

**Figure 6:**
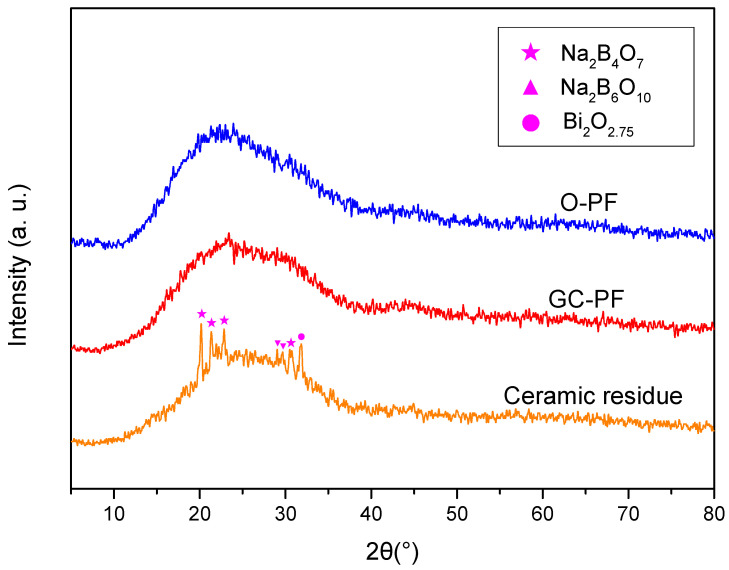
XRD patterns of O-PF, GC-PF (LMG content 50%), and ceramic residue.

In order to further study the phase change of GC-PF ceramization, crystal phases of the foam before and after combustion were analyzed by XRD. As shown in Figure 6, the hump appeared at 13°–35° was amorphous structure of PF [48]. The same amorphous hump appeared in O-PF and GC-PF, however some new diffraction peaks in addition to amorphous hump appear in ceramic residue. The diffraction peaks of borate (Na_2_B_4_O_7_, Na_2_B_6_O_10_) and Bi_2_O_2.75_ appeared in ceramic residue. Borates were generated by B_2_O_3_ and Na_2_O during the combustion of the GC-PF. Bi_2_O_2.75_ was a non-stoichiometric phase of Bi_2_O_3_ after high temperature sintering. XRD patterns confirmed the phase changes of the GC-PF ceramization.

### 3.7. SEM Analysis

**Figure 7:**
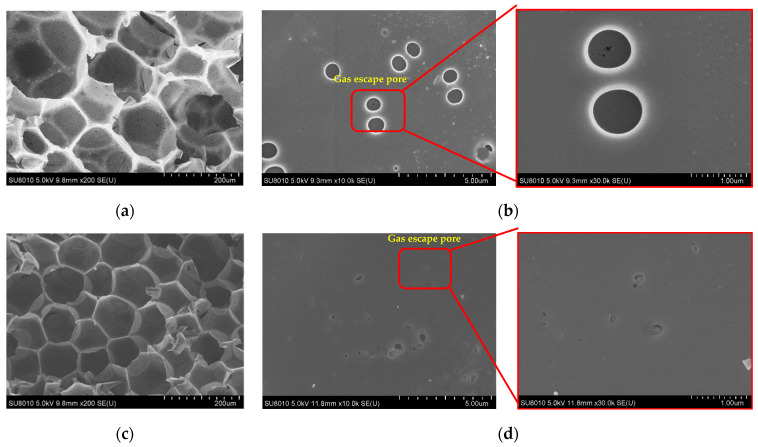
Cell structure of PF: (**a**) and (**b**) O-PF; (**c**) and (**d**) GC-PF (LMG content 50%).

As shown in Figure 7a,c, compared with O-PF, GC-PF (LMG content 50%) showed a more uniform and dense cell structure with fewer cell defects. This may be due to the low potential energy of liquid–solid interface between LMG and phenolic resin matrix, and the nucleation of cells preferentially occurs on the surface of inorganic particles, which increases the cell density and decreases the cell diameter. Therefore, the occurrence of cell rupture of GC-PF due to the expansion of the foaming gas decreased. The volatilization of additives (foaming agent and curing agent) during high curing temperature of PF resulted in gas escape pores on the cell walls, which were then covered and cured again by the uncured resin [49]. Figure 7b,d were the microstructures of the cell walls of O-PF and GC-PF, respectively. There was a large number of pinhole-like damages on the cell wall of O-PF, and a thin film was covered on the gas escape pore. The number of gas escape on the cell wall of GC-PF was significantly reduced. The diameter of pores became smaller, and there were granular fillers in the pores. This is because, in addition to providing a large number of interfaces in the system, LMG also has a certain barrier effect on gas. LMG particles were wrapped in the cell wall and gas escaped pores by phenolic resin matrix, thereby reducing the pinhole-like defects of the GC-PF cell wall.

**Figure 8:**
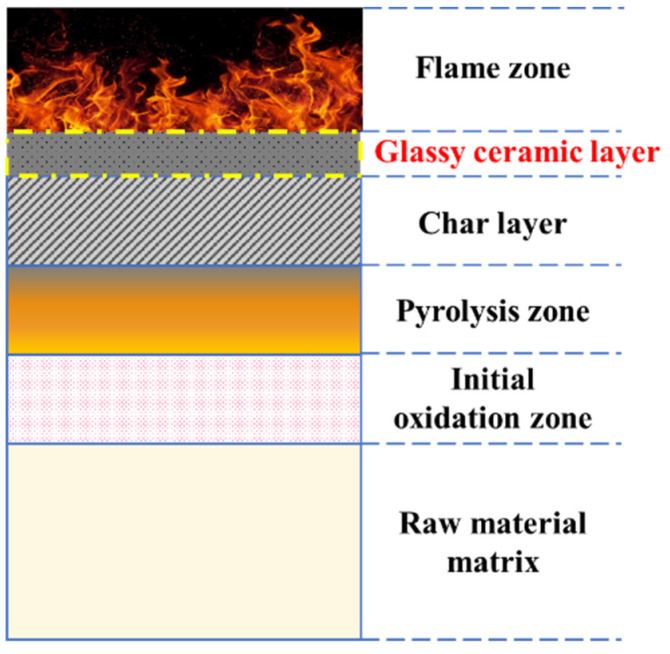
Schematic diagram of GC-PF combustion morphology.

**Figure 9:**
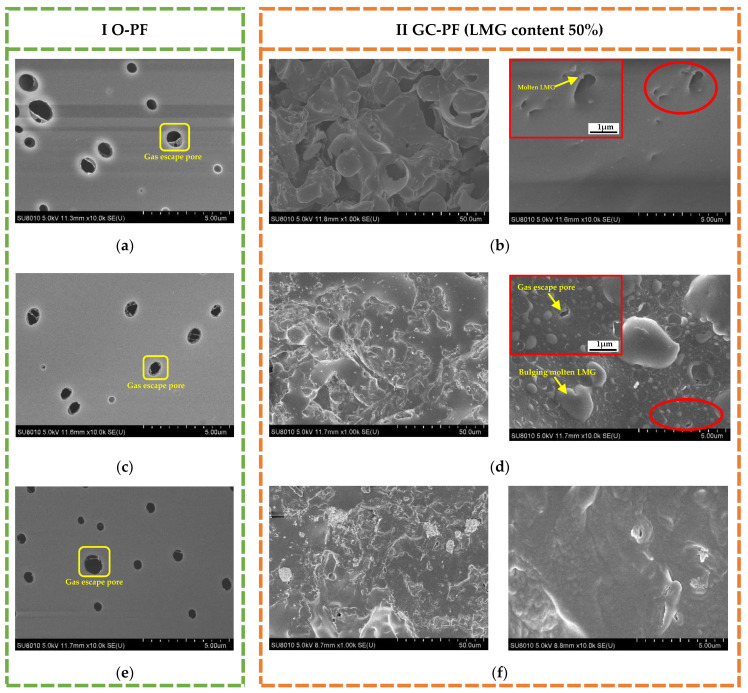
Cell structure of PF after combustion: (**a**,**b**) initial oxidation zone; (**c**,**d**) pyrolysis zone; (**e**,**f**) char layer.

As shown in Figure 8, the non-destructive structure of the raw material matrix of PF was due to the fact that char layer, pyrolysis zone, and initial oxidation zone prevented the further spread of heat. Therefore, when the flame burns on one side of the phenolic foam, the high temperature will not transfer to the other side [50]. Different from the structure of O-PF after combustion, GC-PF formed a new glassy ceramic protective layer on the surface of char layer. As shown in Figure 9 (I O-PF), the closer to the high temperature char layer Figure 9e, the more serious the gas escape pore rupture of O-PF. The rupture of the gas escape pore will allow air and the pyrolytic fuel fragments to flow smoothly in PF, providing comburant for flaming and smoldering of PF. It is inferred that reducing the number of gas escape pores or closing the gas escape pores can effectively improve the flame retardancy of phenolic foams.

**Figure 10:**
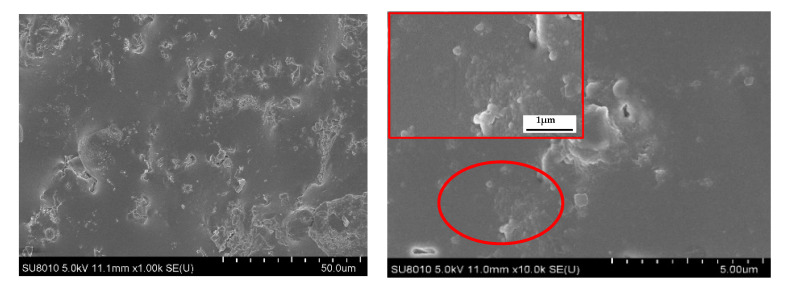
Glassy ceramic layer microstructure of GC-PF (LMG content 50%).

As shown in Figure 9 (II GC-PF), the LMG in the initial oxidation zone began to melt, and a drop-shaped frit appeared near gas escape pores Figure 9b. A large number of bulging bubbles appeared on the cell wall of the pyrolysis zone Figure 9d, which was caused by the bulging of molten LMG near gas escape pores under the action of the gas generated by the pyrolysis of foam. When the gas generated by pyrolysis exceeds the amount that the bubbles can withstand, the bubbles burst, exposing the ruptured gas escape pores on cell wall. Therefore, the formation temperature of glassy ceramics must be lower than that of rapid pyrolysis of polymers [51]. The char layer of GC-PF in Figure 9f no longer showed bulging bubbles, and the char generated by the combustion of the foam was mixed with the molten LMG. As shown in the Figure 10, the glassy ceramic of GC-PF (LMG content 50%) formed a continuous and dense microstructure. The blends may accumulate on the surface of the cell wall. Because of the reduction of the number of gas escape pores and the sealing of gas escape pores after GC-PF ceramization, GC-PF can self-extinguish without smoldering after combustion is complete.

### 3.8. Thermogravimetric Analysis

**Figure 11:**
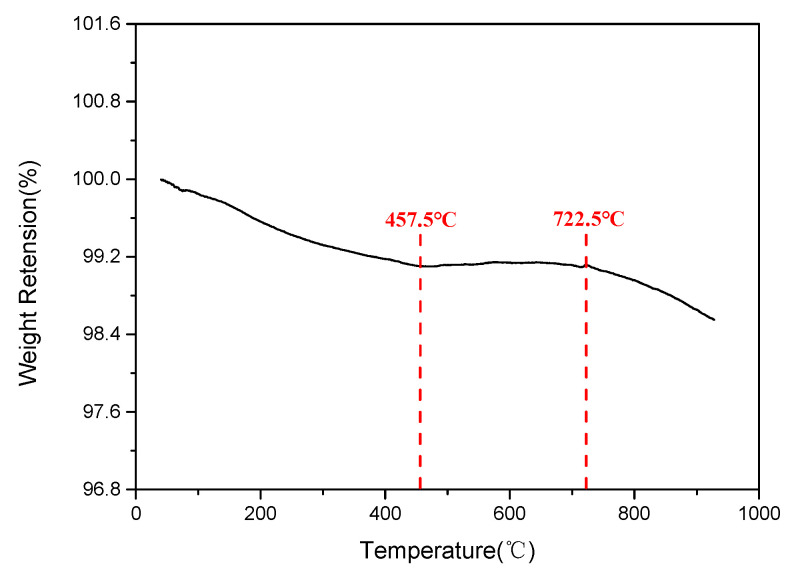
TGA curve of LMG.

As shown in Figure 11, the degradation weight loss rate of LMG was very low, with a total weight loss of 1.5% at 900 °C. The first step of degradation of LMG was the pyrolysis of water molecules, P_2_O_5_, K_2_O and other oxides. The oxides (mainly B_2_O_3_) of LMG began to melt at 457.5~722.5 °C and covered on other inorganic particles, which delayed the degradation rate of LMG. During this process, the by-product pyrolysis gas from the reaction of molten B_2_O_3_ with Na_2_O caused a slight increase in the apparent weight of sample. The pyrolysis of LMG continued after exceeding 722.5 °C, but the degradation rate was very low compared with that of PF.

**Figure 12:**
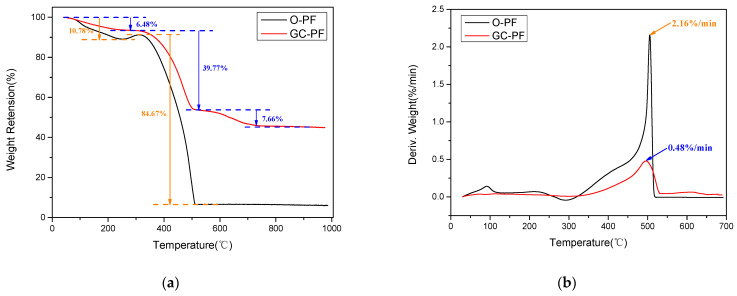
TGA (**a**) and DTG (**b**) curves of O-PF and GC-PF (LMG content 50%).

It could be seen from Figure 12a that the thermal degradation of O-PF consisted of two main steps, and the thermal degradation of GC-PF consisted of three main steps. The first step of degradation of O-PF and GC-PF was the pyrolysis of small molecules such as water molecules, free phenols, free aldehydes, and phenolic hydroxyl groups. The degradation rate of GC-PF did not change significantly on the first step of degradation, while the DTG of O-PF showed a decomposition temperature region (Figure 12b and Table 2). GC-PF exhibited a higher T_5%_ degradation temperature (T_5%_ = 188.3 °C), while O-PF displayed a lower T_5%_ degradation temperature (T_5%_ = 124.9 °C). Compared with O-PF, the T_5%_ degradation temperature of GC-PF was increased by 64 °C. This may be because the reduced level of defect (gas escape pores) in GC-PF cell wall causes the gas generated by pyrolysis can be enclosed in cells. The weight loss of GC-PF at this stage was significantly lower than that of O-PF. The weight increase of O-PF at 251.7~311.9 °C was due to the apparent weight gain of sample caused by the change of buoyancy of a large number of gases generated on the first step of degradation with the increase of temperature.

The temperature region of the second step of degradation of GC-PF was similar to that of the O-PF. The second step of the degradation rate increased significantly, and the main molecular chain began to decompose. As shown in Figure 12a, the weight loss rate of O-PF on the second step of degradation was as high as 84.67%, while that of GC-PF was reduced to 39.77%. GC-PF also showed a lower maximum degradation rate during the second step of degradation (0.48%/min) in Figure 12b, which was 77.8% lower than that for O-PF. This is because the LMG begins to melt when the temperature reaches 450 °C, adhering unmelted inorganic particles and char together to cover the surface of GC-PF to prevent the degradation of the foam at a high rate.

The TGA curve of O-PF was stable at 512.1 °C, while that of GC-PF was stable at 723.8 °C. The third step of the degradation of GC-PF may be due to the sample weight loss caused by the degradation of the by-product during the reaction of molten B_2_O_3_ with the alkaline metal oxide (Na_2_O) to generate borates. GC-PF formed a glassy ceramic protective layer after the third stage of degradation, and the char residue at 900 °C reached 45.25%, which was six times that of O-PF.

### 3.9. DSC Analysis

**Figure 13:**
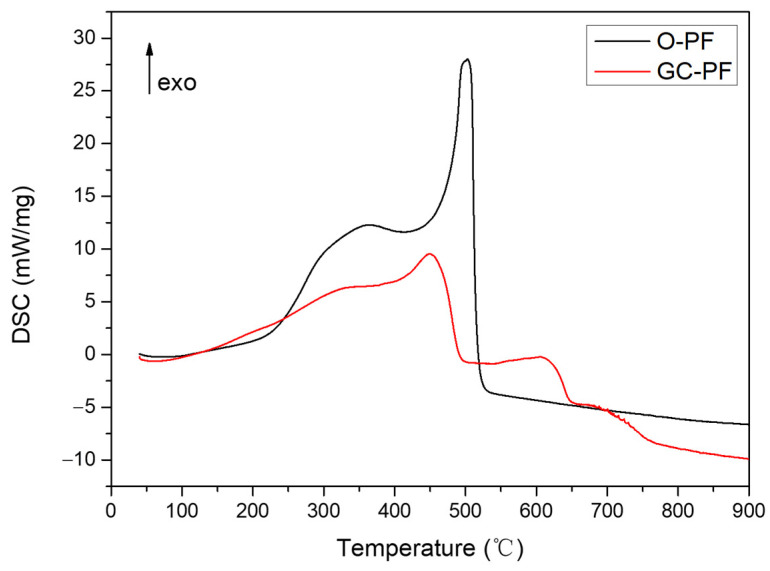
DSC curves of O-PF and GC-PF (LMG content 50%).

The O-PF and GC-PF (LMG content 50%) were characterized by DSC, and the DSC curves are shown in Figure 13. Compared with O-PF, a new exothermic process peak appeared at 540 °C~640 °C in GC-PF. The new exothermic process peak may be attributed to the reaction process of B_2_O_3_ with Na_2_O to generate borates, which is consistent with the third step of thermal degradation. The results indicate that the GC-PF with LMG can form a glassy ceramic protective layer exposed to flame or a high temperature environment.

### 3.10. The Formation Mechanism of Glassy Ceramics Analysis

**Figure 14:**
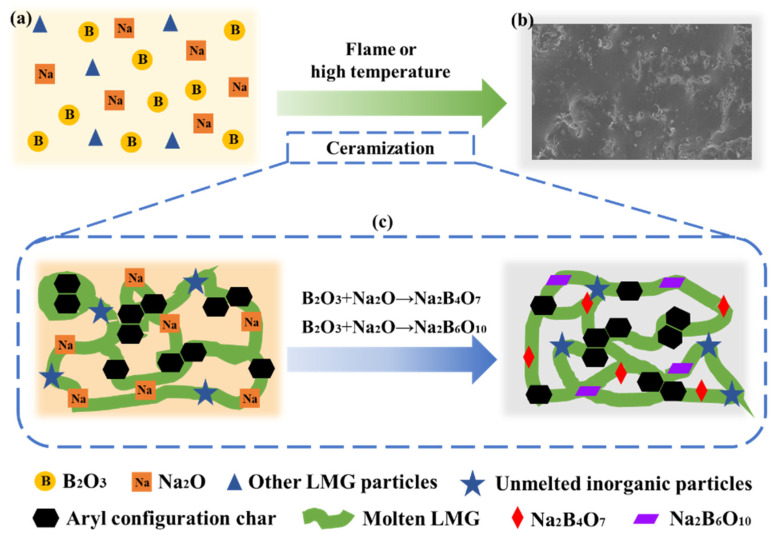
Schematic diagram of the ceramization process of GC-PF: (**a**) GC-PF; (**b**) glassy ceramic layer; and (**c**) ceramization process.

Based on the above results and analysis, a possible mode of formation of glassy ceramics was proposed. As shown in Figure 14, the structure of ceramic protective layer of the GC-PF is different from that of silicon oxycarbide (SiOC) ceramic phase formed by the silica particles [31]. O-PF will be completely pyrolyzed at about 500 °C, and high temperature (above 800 °C) ceramization technology cannot be applied. GC-PF was prepared by incorporation of LMG containing B_2_O_3_ and Na_2_O as the main components into a phenolic resin matrix. B_2_O_3_ is mostly used as a fluxing agent in polymer ceramic composites because of its low melting point temperature (T_m_ = 450 °C). B_2_O_3_ can melt when phenolic foam is not completely pyrolyzed and adhere the unmelted inorganic particles tightly with aryl configuration char produced by GC-PF together. Glassy borates (Na_2_B_4_O_7_, Na_2_B_6_O_10_) are generated when molten B_2_O_3_ contacts with Na_2_O. The structure of boron is transformed from layered structure to cubic structure through free oxygen provided by Na_2_O [52]. The borates are mixed with inorganic particles and aryl configuration char to cover the char layer surface of the GC-PF to form a glassy ceramic protective layer.

## 4. Conclusions

In this paper, a GC-PF with a low ceramization temperature has been formed by incorporation of LMG containing B_2_O_3_ and Na_2_O as the main components into a phenolic resin matrix. FTIR, XRD, TG, DSC, and SEM were used for assessing the changes of GC-PF before and after combustion analysis, indicating that a glassy ceramic protective layer was successfully formed when GC-PF was exposed to flame or a high temperature environment. The glassy ceramic protective layer was a dense structure with a certain self-supporting strength formed by covering the surface of char layer, with blends containing glassy borates generated by the molten B_2_O_3_ and Na_2_O, unmelted inorganic particles, and aryl configuration char. GC-PF exhibited excellent flame retardancy and thermal stability. The LOI of GC-PF was as high as 45.6%, and the char residue at 900 °C was six times greater than that for O-PF. The presence of LMG not only reduces the level of defects in the phenolic foam cell wall (gas escape pore), but also promotes the generation of a glassy ceramic protective layer that could inhibit heat feedback from the combustion zone and reduce the rate of formation of volatile fuel fragments. This paper broadens the research direction of flame retardant of phenolic foam, which can provide a certain basis and reference for the ceramization flame retardant of foam materials.

## Figures and Tables

**Table 1:** Preparation formula of GC-PF.

Samples	Resole Resin (phr ^a^)	LMG (phr)	Surfactant (phr)	Foaming Agent (phr)	Curing Agent (phr)
O-PF ^b^	100	-	6	10	15
GC-PF ^c^-1	100	20	6	10	15
GC-PF-2	100	30	6	10	15
GC-PF-3	100	40	6	10	15
GC-PF-4	100	50	6	10	15
GC-PF-5	100	60	6	10	15

^a^ phr means the parts per hundred of resin by weight. ^b^ O-PF means the ordinary phenolic foam. ^c^ GC-PF means the ceramifiable phenolic foam.

**Table 2:** Detailed TG and DTG analysis of O-PF and GC-PF (LMG content 50%).

		O-PF	GC-PF
TG	T_5%_ * (°C)	124.9	188.3
	residues at 900 °C (wt.%)	6.14	45.25
DTG	First decomposition temperature region (°C)	30~124.9	-
	Second decomposition temperature region (°C)	303.4~519.3	323.1~515.9
	Third decomposition temperature region (°C)	-	535.9~700.0

* Degradation temperature at 5.0 wt.% weight loss.

## Data Availability

The data presented in this study are available on request from the corresponding author.
